# Comparing the Effect of Phenylephrine Bolus and Phenylephrine Infusion for Maintaining Arterial Blood Pressure During Cesarean Delivery Under Spinal Anesthesia: A Randomized Prospective Study

**DOI:** 10.7759/cureus.42713

**Published:** 2023-07-31

**Authors:** Dheer Singh, Jay Brijesh Singh Yadav, Amit K Singh, Mrityunjay K Rai

**Affiliations:** 1 Anesthesiology, Uttar Pradesh University of Medical Sciences, Saifai, IND

**Keywords:** subarachnoid block, phenyephrine, apgar, hypotension, spinal anesthesia, cesarean section

## Abstract

Introduction: Maternal hypotension is a common complication of spinal anesthesia in cesarean section and requires immediate intervention. Phenylephrine is most commonly used as a vasopressor agent for the treatment of hypotension due to subarachnoid block. Our aim was to compare the bolus dose of 50 µg of phenylephrine with a fixed infusion at 50 µg.min^-1^ of phenylephrine for maintaining arterial blood pressure during cesarean delivery.

Materials and method: This was a prospective, randomized comparative study. One hundred normotensive pregnant females aged 18-35 years, body mass index 18-29kg.m^2^, American Society of Anesthesiologists (ASA) physical status classification II scheduled to undergo cesarean section (elective/emergency) under spinal anesthesia were randomly divided into two groups of 50 each. Group PB received a bolus dose of phenylephrine 50 µg after they developed hypotension i.e., a decrease in systolic blood pressure (SBP) ≥ 20% from the baseline. Similarly, patients in Group PI were administered prophylactic infusion using a syringe pump of phenylephrine 50 µg.min^-1^, started just after the administration of subarachnoid block. The phenylephrine infusion was continued either till the delivery of the baby or when SBP rises >20% above the baseline. Parameters like blood pressure, heart rate, and peripheral oxygen saturation were recorded. After the delivery of the baby, the neonatal APGAR score was assessed at one minute and five minutes.

Results: Demographic data were comparable in terms of demographic profile, duration of surgery, and ASA physical status classification between the groups. The heart rate was higher in Group PB compared to Group PI throughout the monitoring period (P<0.001). The fall in mean blood pressure was more in Group PB compared to Group PI till 18 minutes of surgery and was statistically significant (P<0.05). After 18 minutes of surgery, mean blood pressure stabilized and was comparable between the groups. Other variables like APGAR score at one minute and five minutes were comparable between the groups. Bradycardia and hypertension were more common in Group PI whereas hypotension, nausea, and vomiting were more common in group PB.

Conclusion: We concluded that during cesarean section under spinal anesthesia, phenylephrine infusion provides better hemodynamic stability and APGAR score during the perioperative period.

## Introduction

Subarachnoid block is an anesthetic technique of choice for cesarean section [1]. It has certain advantages over general anesthesia such as rapid onset, high success rate, less maternal and fetal side effects with minimal maternal discomfort like difficult intubation, sore throat, and laryngeal injury, etc. Hypotension is common during the induction of spinal anesthesia for cesarean delivery. It is associated with maternal hypotension in 70-80％ of cesarean sections without pharmacological prophylaxis [2].

Maternal hypotension after spinal anesthesia in cesarean delivery is defined as a decrease in systolic blood pressure <100 mm Hg or < 20% from the baseline [3-4]. If hypotension is not corrected quickly, a decrease in blood pressure can have deleterious effects which include nausea and vomiting, lightheadedness, dizziness, and cardiovascular instability as well as a decrease in uteroplacental blood flow with resultant fetal acidosis, hypoxia, and bradycardia [5-6]. The basic components of management are: (1) fluid management like preloading and co-loading, (2) positioning protocols like left lateral position, use of wedge, and use of leg wrapping or sequential compression, etc., and (3) pharmacological agents like phenylephrine, ephedrine, norepinephrine, and mephentermine, etc. [7].

Currently, phenylephrine is considered the vasopressor of choice [8-9]. Phenylephrine is primarily an alpha-1 adrenergic receptor agonist with minimal to no beta-adrenergic activity; therefore, it is ideal for elevating mean arterial pressure. It causes venous and arterial vasoconstriction and increases cardiac preload without having any significant direct effects on cardiac myocytes [10]. Phenylephrine is associated with baroreceptor-mediated bradycardia subsequently leading to a reduction in cardiac output. This incidence has been reported in 30% of cases and it has a negligible effect on healthy pregnant women and fetus. But in high-risk pregnancies like associated maternal cardiac disease, placental insufficiency, and fetal distress, preservation of cardiac output and heart rate might be necessary to mitigate the deleterious effects [11]. Continuous infusion regimens of phenylephrine are suggested that lead to fewer incidences of hypotension compared to a single bolus; however, a stable hemodynamic profile requires a balance between preventing hypotension and avoiding unnecessary hypertension. 

So, we planned to evaluate and compare the effects of phenylephrine bolus and phenylephrine infusion for maintaining stable hemodynamics after subarachnoid block intraoperatively during cesarean section.

## Materials and methods

This was a prospective, randomized, comparative study. After approval from the Ethical Committee of the Uttar Pradesh University of Medical Sciences (Ref no:1887/UPUMS/Dean(M)/Ethical/2020-21 dated 21/06/2021), this study was conducted in the Department of Anesthesia, Uttar Pradesh University of Medical Sciences, Saifai, Uttar Pradesh, India.

Inclusion and exclusion criteria

A total of 100 parturients, aged 18-35 years, with body mass index (BMI) of 18-29 kgm^2^, American Society of Anaesthesiologists (ASA) physical status classification II, cesarean section between 37 to 42 weeks, and singleton pregnancy scheduled for elective or emergency cesarean section under subarachnoid block were included in the study. Exclusion criteria included parturient suffering from any medical illness like cardiopulmonary, hepatorenal, and metabolic disorders, epilepsy, previous brain surgery, history of alcohol and drug abuse, history of allergy to study drugs, spine abnormality, and coagulation abnormality.

Sample size calculation

Based on a previous study, sample size calculation was done considering an alpha error of 5% anFd power of 80% using IBM SPSS Statistics for Windows, Version 20.0 (Released 2011; IBM Corp., Armonk, New York, United States). The total sample size comes out to be 100 patients equally divided into two groups of 50 each.

$n=(Z_{\alpha /2}+Z_{\beta })^{2}\times 2\sigma^{^{2}} /\left ( \mu _{1}-\mu_{2} \right )^{2}$

where n = sample size per group before dropout; Zα/2 = standard normal z-value for significance level α =0.05, which is 1.96; Zβ = standard normal z-value for the power of 80%, which is 0.84; µ1 = mean of affect at baseline; µ2 = mean of effect after drug effect; σ = standard deviation

Pre-anesthetic check-up including a detailed history, general and systemic examination, and laboratory investigations were carried out. Patients were instructed to keep fasting for six hours before surgery and premedicated with tablet ranitidine 150 mg the night before surgery. During emergency cesarean section, patients were premedicated with injection ranitidine 50 mg IV and injection ondansetron 4 mg IV one hour before the surgery.

In the operation theatre, ASA standard monitors were applied to the patients, and heart rate (HR), peripheral saturation of oxygen (SpO2), and non-invasive blood pressure (NIBP) were recorded at baseline. Baseline systolic blood pressure (SBP) was taken as the average of blood pressure measured three times. IV access was obtained with an 18-gauge cannula and patients were administered ringer lactate 15 ml.kg-1 over 10 minutes and thereafter continued at the rate of 10 ml.kg-1.hr-1.

Patients were randomly allocated using a computer-generated number table to either Group PB (Phenylephrine bolus) or Group PI (Phenylephrine infusion). Group PB (n=50) received bolus dose of phenylephrine 50 µg while Group PI (n=50) received an infusion of phenylephrine at the rate of 50 µg.min-1.

Both the anesthesiologist and patients were aware of the assigned groups. Under all aseptic precautions, a subarachnoid block was administered at L4-L5 intervertebral space with 25 G Quincke`s spinal needle in the left lateral position. Injection 0.5% bupivacaine (heavy) 2.0 ml was administered after the free flow of cerebrospinal fluid was seen. The adequacy of the anesthesia effect was assessed.

Patients in Group PB were administered bolus dose of phenylephrine 50 µg IV after a fall in SBP ≥ 20% from baseline and the bolus was repeated if necessary. Similarly, Group PI patients were administered infusion of phenylephrine 50 µg.min-1 prophylactically using a syringe pump, started just after the subarachnoid block. The infusion was continued either till the delivery of the baby or when SBP rises>20% above baseline values.

Assessments

The level of sensory block was assessed by pinprick sensation bilaterally using an alcohol swab. Blood pressure was immediately after administration of the block and then every three minutes until delivery of the baby and subsequently at every three-minute interval till the end of surgery. Other parameters like HR and SpO2 were also recorded. After the delivery of the baby, the neonatal APGAR score was assessed at one minute and five minutes.

The incidence of side effects such as bradycardia (decrease in heart rate less than 60 beats/minute) was treated with 0.5 mg atropine. Shivering was treated with IV injection tramadol 25 mg. Nausea and vomiting were treated with IV 8 mg ondansetron. Pain was treated with injection tramadol 50 mg; however, if pain still persists, it was considered as failed spinal anesthesia, and the patient was administered general anesthesia and excluded from the study.

Statistical analysis

The quantitative data were expressed as mean ± SD and compared between groups using unpaired t-test. Qualitative data were analyzed using Chi-square/Fisher’s exact test. The data was stored in Microsoft Excel spreadsheet (Microsoft Corporation, Redmond, Washington, United States) and statistical analysis was performed using IBM SPSS Statistics for Windows, Version 20.0. P value <0.05 was considered statistically significant.

## Results

**Figure 1:**
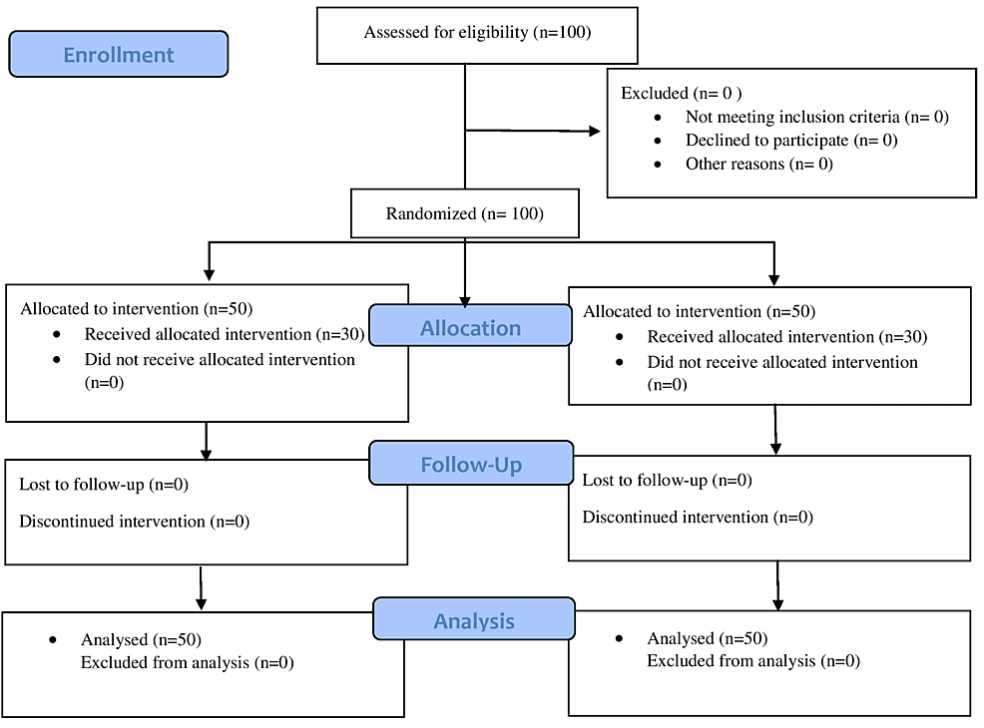
CONSORT flow diagram CONSORT: Consolidated Standards of Reporting Trials

A total of 100 patients were enrolled in the study divided into two groups of 50 each. None of the parturients were excluded from the study as shown in the Consolidated Standards of Reporting Trials (CONSORT) chart (Figure 1).

Demographic profile (age, weight, height, body mass index), ASA physical status classification, and duration of surgery or clinical characteristics were comparable between the groups (p>0.05) (Table 1).

**Table 1:** Distribution of anthropometric parameters between the groups PB: phenylephrine bolus; PI: phenylephrine infusion

	Group PB (n= 50), mean±SD	Group PI (n=50), mean±SD	P-value
Parameters			
Age (years)	24.02±2.69	24.06±2.41	0.469
Weight (kg)	56.22±4.36	55.22±4.14	0.121
Height (cms)	153.10±15.15	154.38±3.24	0.280
BMI (kg/m^2^)	27.72±31.54	23.15±1.34	0.154

**Figure 2:**
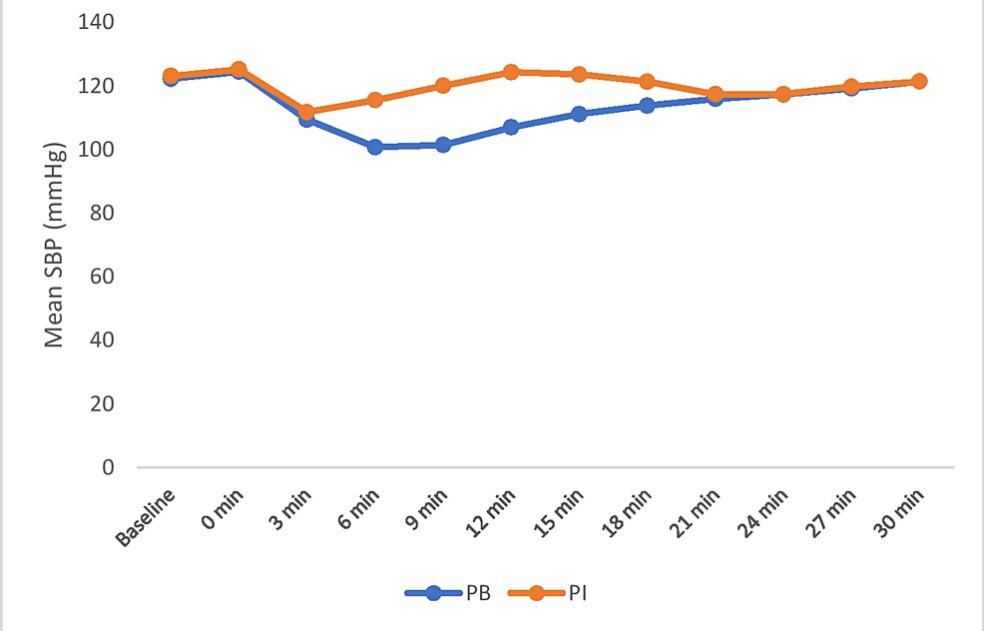
Comparison of systolic blood pressure between the groups. PB: phenylephrine bolus; PI: phenylephrine infusion; SBP: systolic blood pressure

The hemodynamic parameters were monitored and analyzed in both groups. The SBP at baseline and zero minutes were comparable between the groups (P>0.05). During the intergroup comparison, mean SBP remained higher in Group PI compared to Group PB at the sixth minute, ninth minute, 12th minute, 15th minute, and 18th minute and this was statistically significant between the groups (P<0.001). After 18 minutes, mean SBP values were comparable between the groups (Figure 2). 

**Figure 3:**
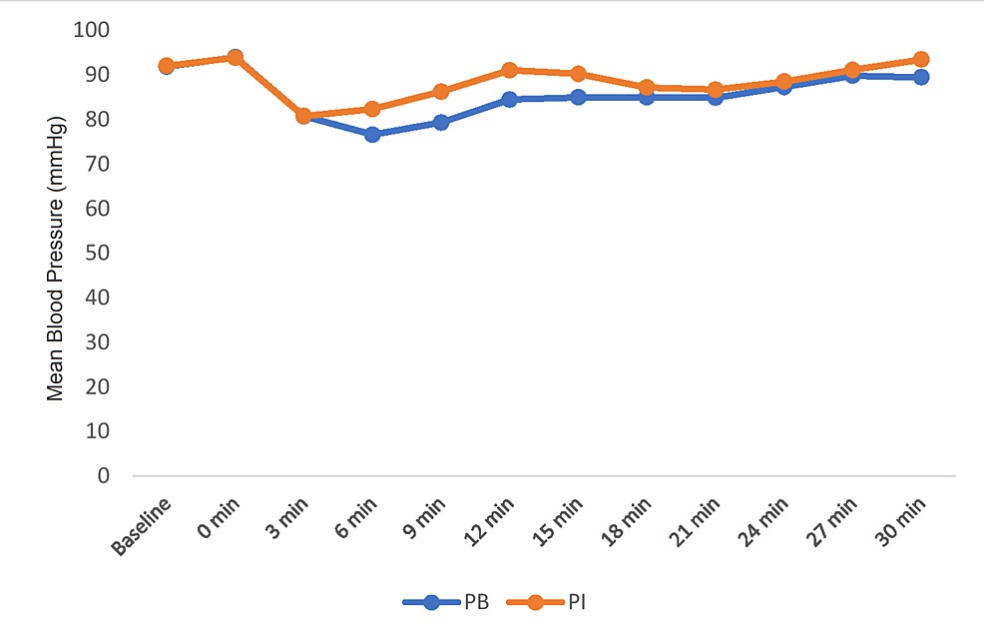
Comparison of mean blood pressure between the groups PB: phenylephrine bolus; PI: phenylephrine infusion

The mean blood pressure at baseline and zero minutes were comparable between the groups (P>0.05). During intergroup comparison, mean blood pressure remained higher in the PI group compared to the PB group at the sixth minute, ninth minute, 12th minute, and 15th minute and were statistically significant between the groups (P<0.001). After 15 minutes, mean SBP values were statistically not significant between the groups (Figure 3).

**Figure 4:**
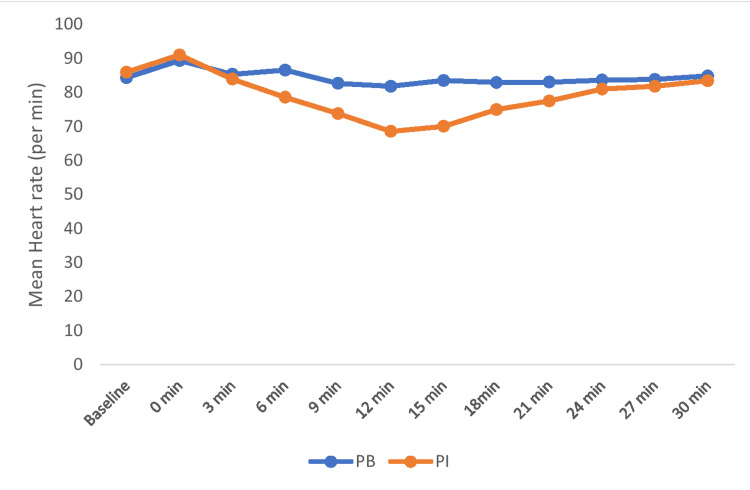
Comparison of heart rate between the groups PB: phenylephrine bolus; PI: phenylephrine infusion

The baseline heart rate at baseline, zero minute, and third minute was comparable between the groups. During intergroup comparisons, mean heart rate values remained higher in Group PB compared to Group PI till 21 minutes during surgery and were statistically significant (P<0.05). After 21 minutes, mean heart rate values were comparable between the groups (P>0.05) (Figure 4).

In Group PB, the fall in SBP >20% from baseline was reported in 50 patients when a 50 µg bolus dose was administered. Six patients required repeated bolus doses of phenylephrine. In Group PI, one patient required a second infusion dose of phenylephrine. The mean total dose of phenylephrine infused was 726 ± 63.28 micrograms till delivery of the baby.

**Figure 5:**
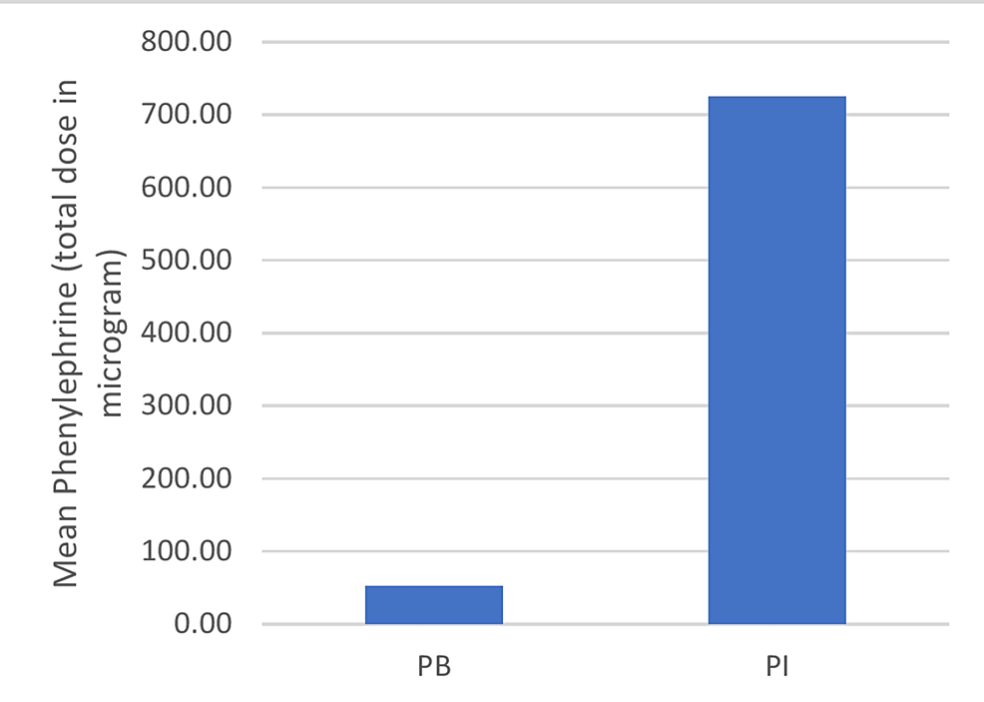
Comparison of phenylephrine dosage required between the groups PB: phenylephrine bolus; PI: phenylephrine infusion

Table 2 shows APGAR score with median and interquartile range between the groups at one and five minutes. During intergroup comparisons, APGAR scores at one and five minutes were recorded as comparable (P=0.136, P= 0.081, respectively).

**Table 2:** Comparison of APGAR score at one and five minutes between the groups IQR: interquartile range

APGAR Score	Phenylephrine bolus (n=50)		Phenylephrine infusion (n=50)		P-value
	median	IQR	median	IQR	
1 min	8.00	(8 - 9)	8.00	(7 - 8)	0.136
5 min	9.00	(9 - 10)	9.00	(9 - 9)	0.081

The incidence of nausea and vomiting in Group PB and Group PI was four out of 50 (8%) and zero out of 50 (0%), respectively. The incidence of bradycardia in Group PB and Group PI was zero out of 50 (0%) and three out of 50 (6%), respectively. The incidence of hypotension in Group PB and Group PI was 50 out of 50 (100%) and one out of 50 (2%), respectively. The incidence of hypertension in Group PB and Group PI was zero out of 50 (0%) and one out of 50 (2%), respectively. On intergroup comparison, statistically significant (p<0.05) difference was seen (Table 3).

**Table 3:** Comparison of side effects between the groups

Side effects	Phenylephrine bolus (PB)		Phenylephrine infusion (PI)		P-value
	n	%	n	%	
Nausea and vomiting	4	8%	0	0%	0.021
Bradycardia	0	0%	3	6%	0.039
Hypotension	50	100%	1	2%	<0.001
Hypertension	0	0%	1	2%	0.157

## Discussion

Spinal anesthesia is a frequently performed procedure for cesarean sections but a disadvantage associated with this technique is the higher incidence of hypotension. As has already been established, maternal hypotension is detrimental to both the mother and fetal outcomes. Although IV fluid preloading is effective in managing subarachnoid block-induced hypotension, it has limited efficacy, which is why vasopressors are needed to manage the hypotension. Various vasopressors are used to manage hypotension but nowadays, phenylephrine is considered as the vasopressor of choice because of greater efficacy, lower placental transfer, and less chances to decrease fetal acidosis.

In our study, the mean SBP was higher in the Group PI as compared to the Group PB and was observed to be statistically significant (p<0.001). A similar study conducted by Chaudhry et al. observed that the mean fall in SBP in phenylephrine bolus (-28.06 ± 5.3 mmHg%) is greater than in phenylephrine infusion (-0.44 ± 4.3 mmHg%) [7]. The result was found to be consistent with our study. Another prospective intervention study conducted by Buthelezi et al. reported that prophylactic phenylephrine infusion significantly reduces the incidence of maternal hypotension as compared with the phenylephrine bolus group (p<0.011) [12]. This was in concordance with our study. Another study performed by Habib et al. reported that the incidence of hypotension is more in the phenylephrine bolus group compared to the infusion group [13]. Single prophylactic phenylephrine bolus was less effective than prophylactic phenylephrine infusion in reducing the incidence of hypotension. Results were found to be in concordance with our study. Allen et al. observed that prophylactic phenylephrine infusion reduces the incidence and severity of maternal hypotension as compared with placebo (p <0.001) [14]. Similar results were also seen in our study. The present study coincides with the study performed by Ngan Kee et al., which showed that phenylephrine infusion decreases the incidence, frequency, and magnitude of hypotension as compared with the control (phenylephrine bolus 100 microgram/minute) group (p <0.0001) [15]. Heesen et al. observed that the relative risk of hypotension was lower in the phenylephrine infusion group as compared to the bolus group (p<0.05) [16]. Similar observations were also noted in our study.

In our study, the mean heart rate was lower in Group PI as compared to Group PB during the recorded time period (p<0.001). This was in concordance with the study conducted by Chaudhry et al. who reported that the mean heart rate was found to be statistically significant among between the study groups and recorded higher in the bolus group when compared to the infusion group throughout the monitoring period (p<0.001) [7]. Ngan Kee et al. observed that the heart rate was significantly lower over time in the infusion group as compared to the phenylephrine bolus group (p<0.001) [15]. This was similar to our study. Habib et al. found that the phenylephrine infusion is associated with an overall lower heart rate as compared with phenylephrine bolus [13]. The result was found to be consistent with our study. In a study conducted by Lee et al., the phenylephrine infusion group showed a significant reduction in heart rate (p<0.05) [8]. Similar observations were also reported in our study. Buthelezi et al. found that the heart rate was recorded lower in the infusion group throughout the monitoring period as compared to the bolus group [12], which was consistent with our result.

In the current study, comparison of APGAR scores at one minute and five minutes for Group PB and Group PI were 8.08±0.70, 9.32±0.47 and 7.92±0.75, 9.18±0.52, respectively, and the difference in the groups was found to be statistically not significant (p>0.05). This is consistent with the study done by Ngan Kee et al., who conducted a study on prophylactic phenylephrine infusion for preventing hypotension during spinal anesthesia for cesarean delivery and found that APGAR scores were statistically comparable during intergroup comparison (p>0.05) [15].

Habib et al. also compared phenylephrine bolus and phenylephrine infusion on maternal hemodynamics and neonatal outcomes in women undergoing cesarean delivery under spinal anesthesia and observed that the APGAR score was statistically not significant difference during intergroup comparison (p>0.05) [13]. The observed finding was similar. In the study conducted by Lee et al., the phenylephrine infusion group showed a statistically not significant (p>0.05) difference in intergroup comparison [8]. Results were found similar to our study. The study by Chaudhry et al. found that APGAR scores at one minute and five minutes compared between the two groups were statistically not significant (p =0.830, 0.254) [7]. We have also found comparable results.

In our study, adverse effects like nausea and vomiting, bradycardia, hypotension, and hypertension were also observed in both groups. Bradycardia and hypertension were reported more in Group PI compared to Group PB. Incidence of hypotension, nausea, and vomiting was more in Group PB as compared to Group PI but results were statistically significant (p<0.05). George et al. observed that the incidence of hypotension-associated nausea and vomiting was significantly (p<0.001) greater in the bolus group (54%) compared to the infusion group (13%) [17]. The incidence of hypotension and the number of hypotensive episodes were significantly (p<0.001) more in the bolus group as compared to the infusion group. Hypertensive episodes were more in the infusion group as compared to the bolus group. (p<0.001) Similar results were also reported in our study. Similar observations were also reported in the study conducted by Ngan Kee et al. who found that there was a less frequent incidence of nausea and vomiting in the infusion group (one of 26; 4%) compared with the bolus group (five of 24; 5%) [15]. They also found that the incidence of hypertension was more frequent in the infusion group compared with the bolus group and the incidence of hypotension and the number of hypotensive episodes were significantly (p<0.001) greater in the bolus group (range 0-12) as compared to the infusion group (range 0-4). The incidence of bradycardia more in the infusion group. A retrospective meta-analysis study conducted by Habib et al. observed that there were lower incidences of nausea, vomiting, and hypotension in the infusion group as compared to the bolus group [13]. They also observed that the incidence of bradycardia was more in the phenylephrine infusion group as compared to the phenylephrine bolus group. A similar observation was seen in our study.

Limitation

The limitation of the current study was that a single bolus dose of 50 microgram phenylephrine was used instead of multiple titrated doses, when compared with continuous infusion. This was done to maintain the simplicity of the procedure and also because continuous intra-arterial blood pressure monitoring was not done.

## Conclusions

From our study, we can conclude that phenylephrine, when given as a prophylactic infusion in doses of 50 µg/minute, leads to greater control of hypotension after spinal anesthesia under cesarean section and decreased intraoperative maternal side effects such as tachycardia, nausea, and vomiting when compared with phenylephrine bolus dose of 50 µgm. The APGAR score and other hemodynamic parameters were found comparable in the two groups.
